# Food allergy alters jejunal circular muscle contractility and induces local inflammatory cytokine expression in a mouse model

**DOI:** 10.1186/1471-230X-9-33

**Published:** 2009-05-18

**Authors:** Jørgen Valeur, Jani Lappalainen, Hannu Rita, Aung Htun Lin, Petri T Kovanen, Arnold Berstad, Kari K Eklund, Kirsi Vaali

**Affiliations:** 1 Institute of Medicine, University of Bergen, Bergen, Norway; 2 Department of Medicine, Haukeland University Hospital, Bergen, Norway; 3 Wihuri Research Institute, Helsinki, Finland; 4 Faculty of Agriculture and Forestry, Statistics and Methodology, University of Helsinki, Helsinki, Finland; 5 Department of Rheumatology, Helsinki University Central Hospital, Helsinki, Finland

## Abstract

**Background:**

We hypothesized that food allergy causes a state of non-specific jejunal dysmotility. This was tested in a mouse model.

**Methods:**

Balb/c mice were epicutaneously sensitized with ovalbumin and challenged with 10 intragastric ovalbumin administrations every second day. Smooth muscle contractility of isolated circular jejunal sections was studied in organ bath with increasing concentrations of carbamylcholine chloride (carbachol). Smooth muscle layer thickness and mast cell protease-1 (MMCP-1) positive cell density were assayed histologically. Serum MMCP-1 and immunoglobulins were quantified by ELISA, and mRNA expressions of IFN-γ, IL-4, IL-6 and TGFβ-1 from jejunal and ileal tissue segments were analyzed with quantitative real-time PCR.

**Results:**

Ovalbumin-specific serum IgE correlated with jejunal MMCP-1^+ ^cell density. In the allergic mice, higher concentrations of carbachol were required to reach submaximal muscular stimulation, particularly in preparations derived from mice with diarrhoea. Decreased sensitivity to carbachol was associated with increased expression of IL-4 and IL-6 mRNA in jejunum. Smooth muscle layer thickness, as well as mRNA of IFN-γ and TGF-β1 remained unchanged.

**Conclusion:**

In this mouse model of food allergy, we demonstrated a decreased response to a muscarinic agonist, and increased levels of proinflammatory IL-6 and Th2-related IL-4, but not Th1-related IFN-γ mRNAs in jejunum. IgE levels in serum correlated with the number of jejunal MMCP-1^+ ^cells, and predicted diarrhoea. Overall, these changes may reflect a protective mechanism of the gut in food allergy.

## Background

Diarrhoea can be regarded as a protective behaviour of the gastrointestinal tract – a defensive mechanism for elimination of harmful luminal substances. The symptom is rather unspecific, and is caused by a number of different stimuli, including toxins, microbes, parasites, allergens, and even stress. Activation of an innate alarm program, governed by the enteric nervous system, has been proposed as a final common pathway [1]. Whenever a stimulus is perceived as harmful, this pre-programmed alarm system is turned on, and the actions of the gastrointestinal effector tissues (glands, vasculature and musculature) are coordinated in a stereotypical defensive response. Although the relationship between intestinal allergy and motility is fairly complex and poorly understood [2-4], the enteric alarm system is conceivably intricately implicated. This is supported by the fact that exposure to luminal allergen induces a state of proximal small intestinal hyperreactivity that may last for at least 8 hours [5,6]. It is therefore reasonable to assume that the motility changes seen in intestinal allergy will resemble those seen in other defensive states.

Rodent parasite infection models have been used as models of irritable bowel syndrome (IBS). Collins *et al*. developed rat and mouse parasite infection models for studying intestinal contractility *in vitro *in response to carbachol, a stable derivative of acetylcholine [7]. The mechanism of intestinal dysmotility in these models has been shown to involve increased local interleukin (IL) 4 expression, that increases the muscarinic receptor's affinity for its substrate [8]. Interferon gamma (IFN-γ) has been shown to decrease the muscarinic receptor's affinity for carbachol [8]. Other cytokines, such as transforming growth factor beta-1 (TGF-β1) and IL-13, have also been shown to affect the affinity and contractility [8,9]. Work by Vallance *et al*. [10] suggested that local overexpression of IL-4 could induce longitudinal muscle hypercontractility, and transfection with lacZ vector expressing IL-4, but not IL-5, increased carbachol-induced longitudinal muscle contractility when mouse jejunum was studied in smooth muscle organ bath.

Our murine model of intestinal allergy [11], employing no immunostimulating adjuvant, provides an excellent opportunity to study the gastrointestinal motility in IgE-mediated food allergy. To test the hypothesis that intestinal allergy and parasite infection share important pathophysiological features, we have studied jejunal circular muscle contractility in response to carbachol and local cytokine expression levels in jejunum and ileum in our food allergy model.

## Methods

### Sensitization, challenge, and treatment protocols

**Figure 1:**
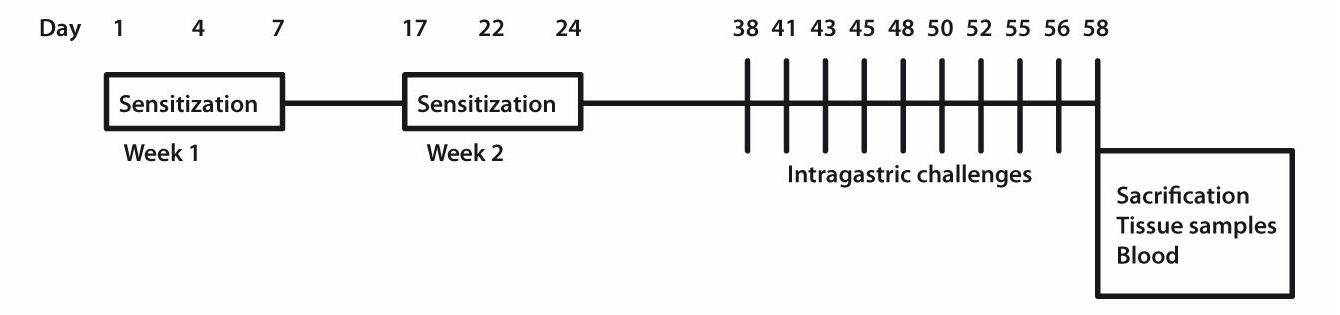
**Timetable for the sensitizations and challenges in the study**. Epicutaneous sensitizations were performed twice in one week intervals, followed by an immunological maturation period. Intragastric (i.g.) administrations (50 mg/mL of ovalbumin) were started on day 38, and repeated 10 times. The mice were sacrificed on day 58, 60 min after the last i.g. administration.

Seven-week old female Balb/c mice were purchased from the National Laboratory Animal Center from Taconic (Taconic, Lille Skensved, Denmark). Mice were maintained under specific pathogen free conditions and on ovalbumin-free diet. After a two week acclimatization period, mice were epicutaneously sensitized with 100 μg of ovalbumin (fraction V, Sigma, St. Louis, MO) in 100 μL of saline, or sham-sensitized with saline (controls). The epicutaneous sensitization was performed according to Vaali *et al*. [11](11), with minor modifications. Briefly, mice were anesthetized with 4% isoflurane (Isoba, Schering-Plough Brussels, Belgium), the back of the mouse was shaved by an electrical razor (Moser Chromo Mini, Wahl GmbH, Unterkirnach, Germany) and the skin was lightly abraded by taping it 4 times with injection tape (Tegaderm™, 3 M, Health Care, Borken, Germany). On days 1 and 4, ovalbumin or saline was placed on a patch of sterile gauze (1 × 1 cm), which was secured to the skin of the mouse back with tape. On days 17 and 22, an identical patch was reapplied to the same site of the skin. Starting from day 38, all mice were challenged 10 times with intragastric (i.g.) doses of ovalbumin (50 mg/mL, 200 μL) (Figure 1).

### Evaluation of fecal samples

Stool consistency was evaluated for diarrhoea at 0, 20, 40, and 60 min after each i.g. dose.

### Tissue preparations

Mice were placed into a carbon dioxide chamber and after loss of consciousness, they were sacrificed with cervical dislocation, and their hearts were punctured. The entire small intestine from pylorus to cecum was excised and divided into two pieces of equal length. The samples for jejunal histology were collected from the beginning of the distal half, the following 2–3 cm segment was prepared for organ bath measurements, and the next 5 mm segment for mRNA measurements. A segment 2.5 cm proximally from the ileocecal junction was collected for mRNA analysis.

### Measurements of smooth muscle contractility in smooth muscle organ bath

Seven millimetre long segments of the small intestine were cut and mounted by hanging from triangle hooks. The hooks were connected to transducers from the upper end, and were inserted through the gut lumen from the lower end, letting the circular muscle to contract. Chambers of 20 mL size containing Krebs-Ringer buffer of the following composition (mM) were used: 119 NaCl, 25 NaHCO_3_, 11.1 glucose, 1.6 CaCl_2 _× H_2_O, 4.7 KCl, 1.2 KH_2_PO_4_, 1.2 MgSO_4 _× 7 H_2_O. The tissue segments were constantly aerated with a mixture of 95% oxygen and 5% of carbon dioxide, and the buffers were kept at 37°C using an external water bath. The initial tension load was set between 0.5–1.0 g, from which the segments spontaneously relaxed over time, and this resting tone was adjusted upon need. The segments were allowed to stabilize for 30 min before the contracting agonist was administrated. Each concentration of carbachol (Sigma) was allowed to affect for approximately 2–3 min, and the contraction curve of carbachol (0.01 μM – 33 μM) was drawn. The contractions were measured with isometric transducers, connected to amplifiers, and recorded with measurement software IOX Version 1.7 (EMKA Technologies, Paris, France). After the experiments, the tissue segments were carefully removed from the hooks, air dried for some weeks, and weighted. The results were expressed as tension in mg/mm^2^, which is the contraction divided by cross-sectional area, CSA (CSA = tissue dry weight/(tissue length × tissue density); assuming that tissue density was 1.05 mg/mm^3 ^[12]). The contractions were compared at each concentration levels. In addition, the allergic and control mice were compared with respect to the average slope of tension as a function of logaritmic carbachol-concentration. The slope was calculated separately for each mouse using the carbachol-concentrations that started with the first non-zero tension value and ended at the last concentration that was shown effective for increasing tension.

### MMCP-1 and ovalbumin-specific IgE, IgG_1 _and IgG_2A _ELISA measurements

MMCP-1 levels were quantified by ELISA according to the manufacturer's instructions (Moredun Scientific Ltd, Midlothian, UK), and all the samples were diluted 1:1000 and 1:5000. The antibodies used in the study were purchased from Becton Dickinson (San Diego, CA). For assays of serum ovalbumin-specific IgE, rat anti-mouse IgE (clone R35–72, 2 μg/mL) was used for coating in 50 mM NaHCO_3 _(pH 9.6) overnight at 4°C, and the serum samples were diluted 1:20 and 1:50 in PBS. For ovalbumin-specific IgG_1 _and IgG_2A _ELISAs, the plates were coated with ovalbumin (fraction V, Sigma, 2 μg/mL), and the serum samples were diluted 1:10 000 and 1:30 000 for IgG_1_, and 1:50 and 1:200 for IgG_2A _in PBS. For the detection of specific IgG_1 _and IgG_2A_, a commercially available biotin-labeled anti-mouse isotype-specific secondary antibody was used at a concentration of 2 μg/mL (A85-3 MoAb, BD Pharmingen, San Jose, CA). For ovalbumin-specific IgE detection, ovalbumin was biotinylated according to manufacturer's instructions (EZ-Link™ Sulpho-NHS-Biotinylation Kit, Pierce, Rockford, IL) and the excess label was removed by dialyzing with Slide-A-Lyzer Dialysis Cassette (Pierce). For total IgE detection, biotinylated secondary antibody was used (R35–118, BD Pharmingen). The optimal working dilution (1:1000) of the biotinylated ovalbumin was estimated by serial dilution of the samples in ELISA. Streptavidin-horseradish peroxidase (BD PharMingen) and peroxidase substrate reagents (Kirkegaard & Perry Laboratories, Gaithersburg, MD) were used for immunodetection, and the results were expressed as absorbance at 405 nm wavelength.

### Histology

Jejunal specimens were embedded in 4% formaldehyde solution and after paraffin embedding, specimen were sectioned to 5 μm onto superfrost glasses (Erie Scientific Company, Portsmouth, NH). For the major subtype of intestinal mast cells, a MMCP-1 rabbit HRPO-conjugated antibody (Moredun Scientific Ltd) was used as previously described [11]. Briefly, the specimens were first deparaffinized, followed by antigen retrival using microwave heating, and endogenous peroxidase activity was blocked with 5% H_2_O_2_. Next, HRPO-conjugated rabbit anti-mouse MMCP-1 primary antibody was used with diaminobenzidine tetrachloride (DAB) (Vector Laboratories, Burlingame, CA). Hematoxylin background staining was performed with Fluka's solution according to Mayer (Sigma-Aldrich Chemie GmbH, Steinheim, Germany). For the determination of MMCP-1^+ ^cell density, a minimum area of 0.5 mm^2 ^from a transsectional segment was examined.

Smooth muscle thickness was measured under light microscope (Olympus BX51) using Olympus Soft Imaging Solutions version 2.6 (Münster, Germany). The assessment was performed twice in a blinded manner, and the average values were reported.

### Analysis of gene expression

Tissue samples from jejunum and ileum were homogenized in TRIzol reagent (GIBCO-BRL, Paisley, UK) using an Ultra-Turrax T8 homogenizator (IKA Labortechnik, Staufen, Germany). Total RNA was isolated according to the manufacturer's instructions (GIBCO) and redissolved in DEPC-treated water. Nucleotide concentrations were determined by NanoDrop ND-1000 spectrophotometer (NanoDrop Inc, Wilmington, DE). The RNA samples were converted into their respective cDNAs using 3 μg of RNA by moloney murine reverse-transcriptase (GIBCO-BRL) and oligo(dT)_15 _primers (Promega, Madison, WI) for 55 min at 37°C. For real-time quantitative PCR, the pre-designed primers and fluorogenic TaqMan probes for (IL-4, IL-6, IFN-γ, TGF-β1, and β-actin) were purchased from PE Applied Biosystems (PE Applied Biosystems, Foster City, CA). The samples were amplified in duplicate with ABIPrism 7500 Sequence Detector System (PE Applied Biosystems) using the following program: a 2 min incubation at 50°C for uracil-N-glycosylase (UNG) treatment, followed by a 10 min pre-incubation at 95°C, and 45 cycles consisting of denaturation for 15 sec at 94°C, and 1 min at 60°C for primer annealing and polymerase extension. The data were normalized relative to the expression of β-actin by applying the introduced algorithm (the 2^-ΔΔCT ^method) [13].

### Ethical permissions

The research plan was approved by the by the Norwegian Animal Research Authority and conducted according to the European Convention for the Protection of Vertebrates Used for Scientific Purposes.

### Statistics

Values were compared using non-parametric statistics (Mann-Whitney's U-test) while slopes of contractility responses with respect to carbachol concentrations were compared using parametric statistics (Student's t-test). Correlation analyses were performed using the Spearman's rank correlation coefficient. Data are presented as mean ± standard deviation (SD) unless otherwise stated. Values of *P *< 0.05 were considered statistically significant. Calculations were performed with GraphPad Prism 5 program (GraphPad Software, Inc. San Diego, CA) or SPSS (version 14, SPSS Inc., Chicago, IL).

## Results

### Most of the ovalbumin-sensitized and -challenged mice have diarrhoea and increased density of MMCP-1^+ ^mast cells in jejunum

None of the mice in the control group had diarrhoea, whereas 5 of the 8 mice in the allergen-sensitized and -challenged group had diarrhoea. The diarrhoea occurred after the 6^th ^i.g. dose, and thereafter repeatedly 20 to 60 min after each i.g. dose. Mice from the ovalbumin-sensitized and -challenged group had significantly increased levels of serum ovalbumin-specific IgE, IgG_2A _and IgG_1 _antibodies, when compared to the control group, *P *< 0.01 (Table 1).

**Table 1:** Ovalbumin-specific serum immunoglobulin levels in ovalbumin- and sham-sensitized mice.

	ova-sp. IgE			ova-sp. IgG_2A_			ova-sp. IgG_1_		
EC	Dilution	Mean	SD	Dilution	Mean	SD	Dilution	Mean	SD
Saline	**1:20**	0.121	0.017	**1:50**	0.485	0.463	**1:10000**	0.205	0.113
Ova	**1:20**	0.689 **	0.292	**1:50**	0.826	0.574	**1:10000**	2.449 **	0.205
Saline	**1:50**	0.167	0.053	**1:200**	0.150	0.053	**1:30000**	0.140	0.075
Ova	**1:50**	0.385 **	0.139	**1:200**	0.370 **	0.198	**1:30000**	1.575 **	0.521

Results represent optical density values at 405 nm, measured from serum samples obtained after sacrification of the animals. Ova-sp = ovalbumin-specific; EC = epicutaneous sensitization; ova = ovalbumin-sensitized mice, (*n *= 8); saline = sham-sensitized mice, (*n *= 5); * = *P *< 0.05, ** = *P *< 0.01 (Mann-Whitney's U-test); SD = standard deviation. All samples were assayed in duplicates.

**Figure 2:**
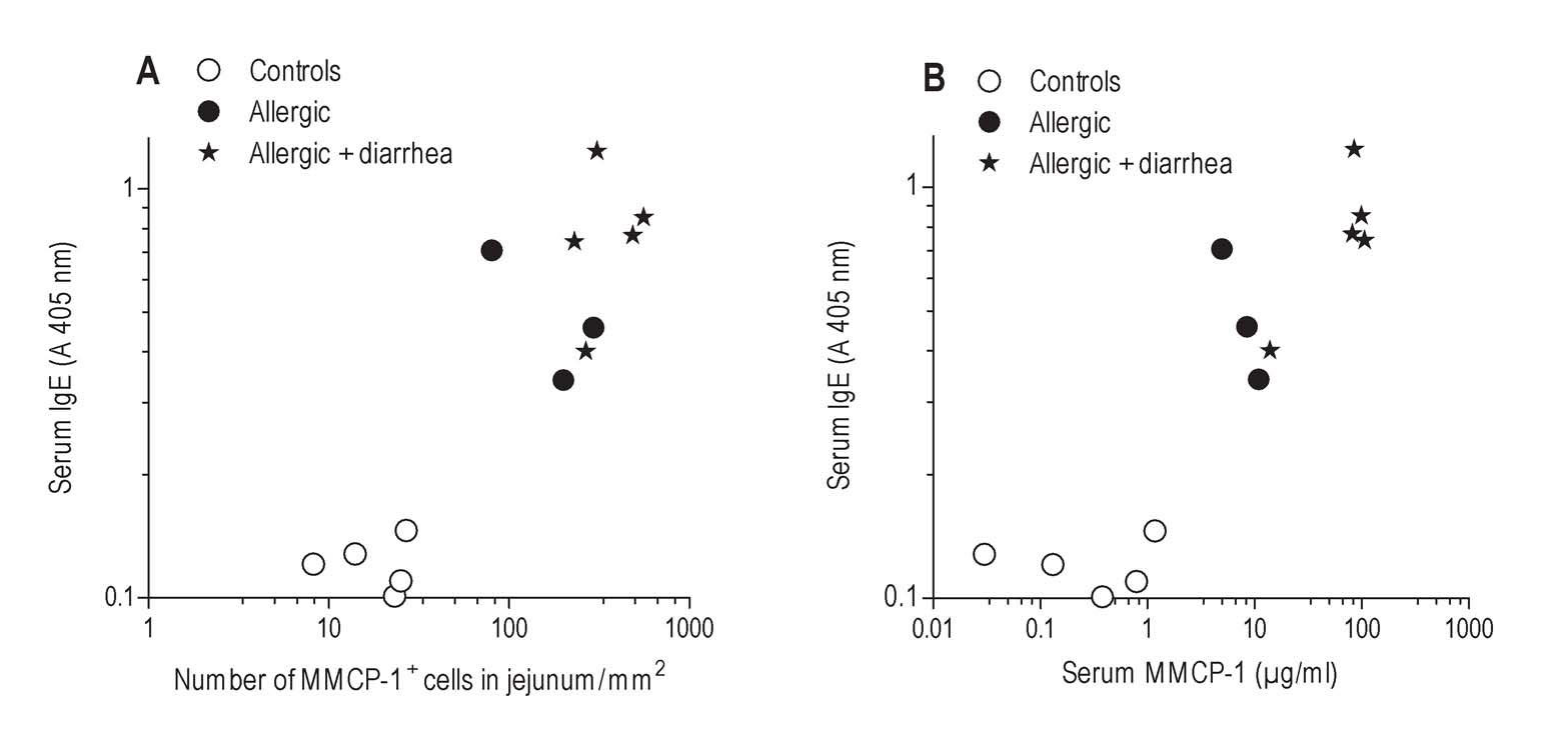
**Correlation of jejunal MMCP-1^+ ^cell density (A) and serum MMCP-1 (B) with serum ovalbumin-specific IgE**. The results are shown for the sham- (*n *= 5) and ovalbumin sensitized mice (*n *= 8), all of which were challenged with ovalbumin. The mice with (*n *= 5) and without diarrhoea (*n *= 3) are indicated separately. All samples were assayed in duplicates.

The density of the jejunal MMCP-1^+ ^cells in the total calculated area correlated with the serum levels of ovalbumin-specific IgE (Spearman's *r *= 0.879, *P *< 0.0001, Fig. 2A). In addition, serum MMCP-1 levels correlated with serum ovalbumin-specific IgE (Spearman's *r *= 0.863, *P *< 0.0001, Fig. 2B).

### The food-allergic mice with diarrhoea have altered jejunal circular smooth muscle contractility towards carbachol

**Figure 3:**
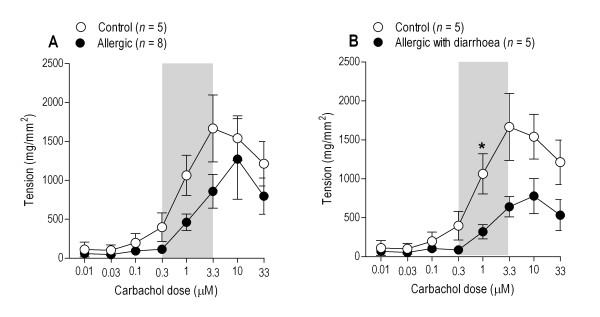
**Cumulative concentration-response curves for carbachol-induced contractions of jejunal circular muscle preparations**. Contractions are expressed as tension, i.e. adjusted with the CSA-formula, using dry tissue weight. A: Mean responses (with SEM) of control (*n *= 5) and allergic mice (*n *= 8) are shown. B: Only the allergic mice with diarrhoea (*n *= 5) are compared with controls. The asterix (*) indicates statistically significant difference (Fig. B: *P *= 0.016) between the two groups at the respective carbachol concentration (Mann-Whitney's U-test). To evaluate differences in response to carbachol, slope of the dose-response curve was calculated for each animal for carbachol concentrations ranging from 0.3 to 3.3 μM (shaded area). The mean slope values differed significantly between the groups only when allergic mice with diarrhoea (n = 5) were compared with the control mice (Fig. B: *P *= 0.048).

Cumulative doses of carbachol induced contractions in the small intestinal segments in a concentration-dependent manner up to 3.3 μM. Higher concentrations resulted in tachyphylactic responses. The circular intestinal segments from control mice contracted with higher potential than those from allergic mice (Fig. 3), but mean values were significantly different only in response to 1 μM carbachol (*P *= 0.016; Mann-Whitney's U-test) when allergic mice with diarrhoea (*n *= 5) and controls (*n *= 5) were compared. The mean slopes of the 0.3 to 3.3 μM carbachol-response curves for these groups were also significantly different (*P *= 0.048; Fig. 3B).

### Intestinal smooth muscle thickness is not increased in food-allergic mice

**Figure 4:**
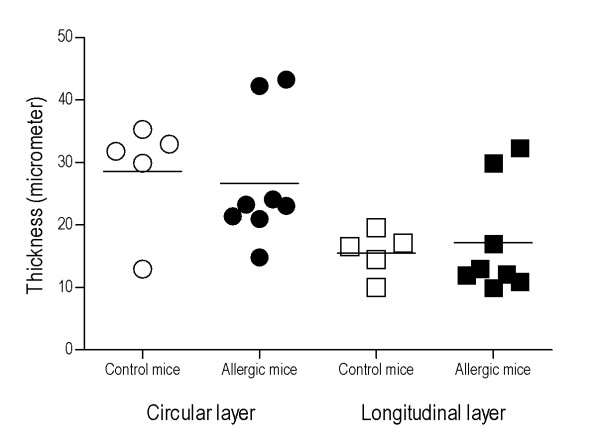
**Comparison of smooth muscle layer thickness in jejunal segments**. No difference was found neither in longitudinal nor circular layer thickness between allergic mice (*n *= 8) and controls (*n *= 5). Mean values are indicated as lines. The assessment was performed twice in a blinded manner.

There was no difference in the jejunal circular or longitudinal smooth muscle layer thickness between allergic and control mice, when measured in the formalin-fixed, paraffin-embedded tissue section (Fig. 4).

### The expression of inflammatory cytokines in jejunum, but not in ileum, were increased in food-allergic mice

**Figure 5:**
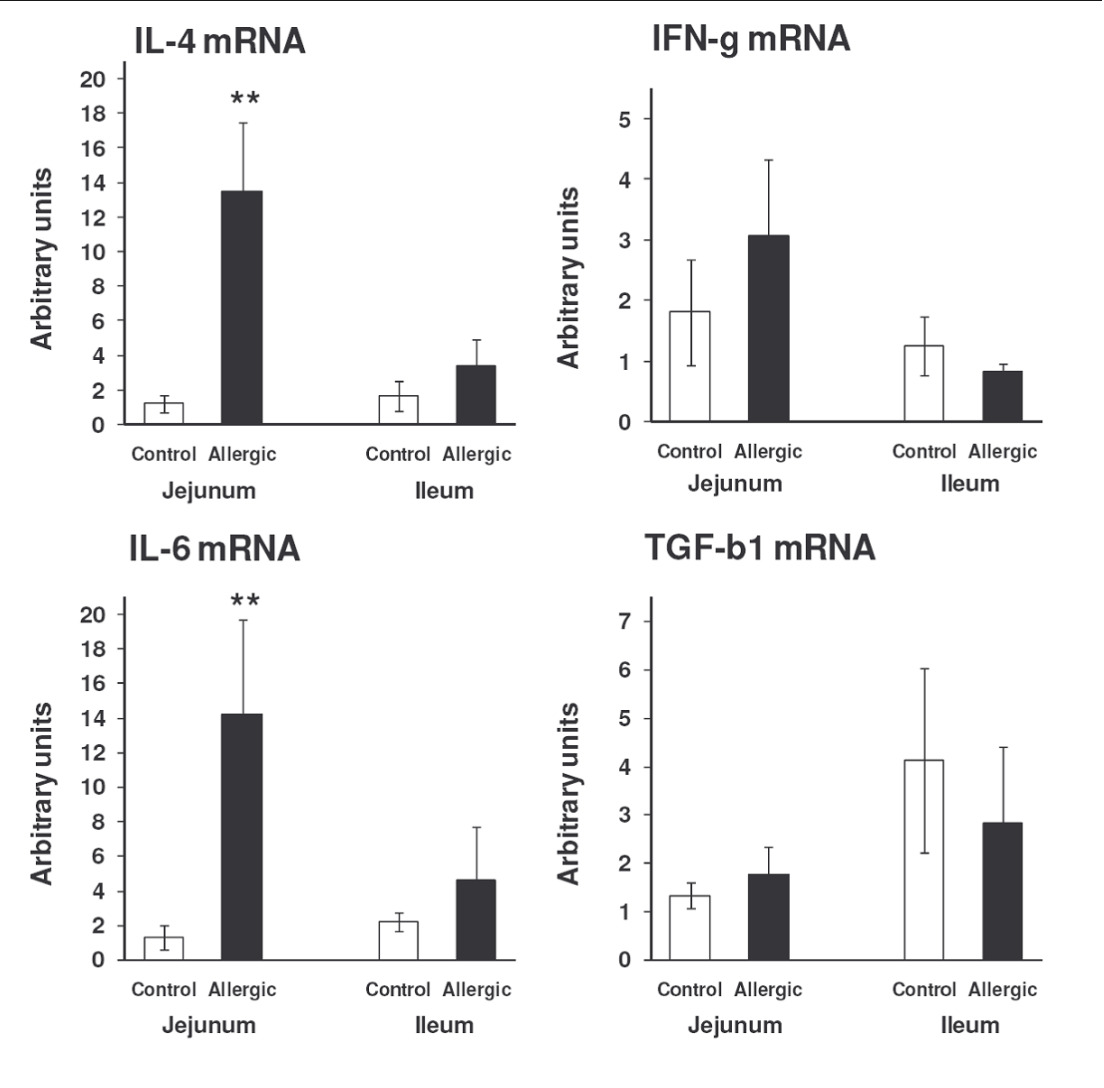
**Expression of cytokine mRNAs in jejunum and ileum**. When compared to control mice (*n *= 5) the expression of IL-4 and IL-6, but not IFN-γ or TGF-β1, were significantly increased in allergic mice (*n *= 5–8). Data are presented as means ± SEM. Mann-Whitney's U-test: **P < 0.01. All samples were assayed in duplicates.

The mRNA expression levels of the cytokines IL-4 and IL-6 were significantly increased in the jejunum of the food allergy animals (*P *= 0.002 and *P *= 0.005, respectively). In contrast, mRNAs levels of the cytokines IFN-γ and TGF-β1 were not significantly different between allergic and control mice (*P *= 0.177 and *P *= 0.435, respectively) (Fig. 5). In the ileum, no significant differences were found in the expression of the studied cytokines between the allergic and control group (Fig. 5).

## Discussion

In our model of food allergy, the allergic mice were found to have jejunal circular smooth muscle hypocontractility in response to submaximal concentrations of the acetylcholine mimetic carbachol. Moreover, the mice with food allergy had increased levels of Th2-related IL-4 and pro-inflammatory IL-6 cytokine mRNAs in the jejunum, whereas the thickness of jejunal muscular layers was not affected. We also found a strong positive correlation between the number of mast cells and ovalbumin-specific IgE.

Only contractility of the longitudinal layer has been assessed in most of the studies investigating intestinal smooth muscle function in parasite infection models. The early results from Vermillion *et al*. [14] showed development of hypercontractility of the longitudinal layer. However, the circular layer seems to behave differently, and has shown hypocontractility in response to muscarinic agonists [15,16]. In a study using TNBS-induced colitis in ileum of guinea pig [17], opposite responses of circular and longitudinal smooth muscle segments to carbachol were observed. It is thus possible that in the presence of inflammation, the circular muscle layer will show hypocontractility instead of hypercontractility in response to muscarinic agonists. For effective peristalsis, the two muscle layers require complex collaborative function. But since smooth muscle function is governed by extensive extramural neural circuits, the performance of an isolated bowel segment does not necessarily reflect the performance of this segment *in vivo*.

In this study, we chose to measure contractility of the circular muscle layer, as a previous report suggests a more widespread muscle hypertrophy and hyperplasia in the circular than in the longitudinal layer of jejunum in a parasite infection model [18]. In contrast to the findings in the parasite model, though, we found no evidence of hypertrophy of the smooth muscle layers. Nevertheless, an altered responsiveness to a cholinomimetic, as seen in our allergy model, may well reflect an allergen-induced defence mechanism with a bearing to the disturbed small intestinal motility pattern seen in intestinal anaphylaxis [19] and in patients with food hypersensitivity [20-22]. Interestingly, colonic hypocontractility [23] and thinning of duodenal and colonic smooth muscle layers [24] has been described in other food allergy models.

There are some important differences between the allergy and parasite models. In our model, the allergen-induced stress is short-lived, and the challenges are intermittent, whereas the intestine has to generate force continuously for days to expell a parasite. Conceivably, therefore, a parasite infection may influence more profoundly on muscular mass and the number of receptors. On the other hand, in a *Giardia *infection model [16], no hyperplasia was found, suggesting that this type of infection could have a similar effect on gut motility as food allergy.

Using gene expression analysis, we found significantly elevated levels of IL-4 and IL-6 mRNA in the jejunum of the allergic mice, whereas the expression of IFN-γ was not significantly altered. This is in accordance with the results from parasite infection models [9,25], and also with recent studies in food allergy models [26]. IFN-γ is known for its capability to decrease the muscarinic receptor affinity for carbachol, whereas IL-4, IL-13 and TGF-β1 increase the affinity [8,25,27]. Moreover, we found no difference in the TGF-β1 mRNA expression in jejunum, which is in contrast to the results obtained in the parasite infection models [8]. These results suggest that in our model, the inflammation may not yet have reached a chronic state, such as in the asthmatic airways in which TGF-β1 is known to exert smooth muscle hyperplasia [28]. IL-6, known for its potent immuno-stimulatory and pro-inflammatory effects in inflammatory bowel disease [29], is produced by macrophages, lymphocytes, and intestinal epithelial cells. In colonic cancer, a cross talk between IL-6 and TGF-β has been suggested [30], and in intestinal epithelial cell culture, TGF-β1 may inhibit IL-6 expression [31]. The results suggest an altered Th2/Th1 cell balance in the jejunum, but not in the ileum of our mouse model of food allergy. Interestingly, increased numbers of duodenal IL-4^+ ^cells, but decreased numbers of IFN-γ^+ ^cells, have also been found in mucosal biopsies from the small intestine of patients with non-IgE-mediated food allergy [32].

We found that the number of jejunal MMCP-1^+ ^mast cells, as well as serum levels of MMCP-1, correlated positively with allergen-specific IgE levels, and also with the presence of diarrhoea. These findings highlight the importance of jejunal mast cells and the role of the proximal intestinal tract in food allergy-dependent diarrhoea development. However, whether the T cell-derived cytokines are also important for the development of intestinal pathology cannot be judged by the present results.

## Conclusion

In conclusion, we found evidence of nonspecific small intestinal dysmotility and local changes in inflammatory cytokine expression in a mouse model of IgE-mediated food allergy. Our findings are partly analogous to changes observed in parasite infection models, and implicate that these two conditions activate a common pathway – an enteric alarm system. Although such an activation may be beneficial as an acute defense mechanism, chronic dysmotility may ensue if the system is continuously challenged.

## Competing interests

The authors declare that they have no competing interests.

## Authors' contributions

JV designed the study, carried out the experiments, and prepared the manuscript. JL was responsible for the qPCR analysis and prepared the manuscript. AHL carried out the experiments. HR performed the statistical analysis and prepared the manuscript. PTK and KKE offered financing and facilities for the qPCR analysis, and prepared the manuscript. AB designed and coordinated the study, offered financing, and prepared the manuscript. KV designed the study, taught and carried out the experiments, and prepared the manuscript. All authors read and approved the final manuscript.

## Pre-publication history

The pre-publication history for this paper can be accessed here:

http://www.biomedcentral.com/1471-230X/9/33/prepub
